# Towards automatic home screening of obstructive sleep apnea using combined features from pulse wave amplitude, pulse-to-pulse interval and oxygen desaturation

**DOI:** 10.1007/s11325-026-03592-4

**Published:** 2026-02-19

**Authors:** Wanwara Thuptimdang, Krongthong Tawaranurak, Pattaraporn Panyarath, Wandee Rakim, Katinee Wae-asae, Michael C. K. Khoo

**Affiliations:** 1 Institute of Biomedical Engineering, Department of Biomedical Sciences and Biomedical Engineering, Faculty of Medicine, Prince of Songkla University, Hat Yai, Songkhla Thailand; 2 Department of Otolaryngology Head and Neck Surgery, Faculty of Medicine, Prince of Songkla University, Hat Yai, Songkhla Thailand; 3 Division of Respiratory and Respiratory Critical Care Medicine, Faculty of Medicine, Prince of Songkla University, Hat Yai, Songkhla Thailand; 4 Songklanagarind Hospital Sleep Center, Prince of Songkla University, Hat Yai, Songkhla Thailand; 5 Department of Biomedical Engineering, University of Southern California, Los Angeles, CA USA

**Keywords:** Obstructive sleep apnea, Photoplethysmography, Pulse wave amplitude, Oxygen saturation, Heart rate variability, Respiratory arousal

## Abstract

**Purpose:**

Automatic home OSA screening with pulse oximeters often relies on oxygen saturation (SpO2), which may miss hypopneas associated with arousals. Photoplethysmography (PPG) from pulse oximeters can be processed to extract pulse wave amplitude (PWA) and pulse-to-pulse interval (PPI) which reflect autonomic activation during arousals. However, the role of PWA in automated OSA screening remains unexplored. This study evaluated the added value of PWA for detecting OSA segments, and estimating AHI compared with SpO2 and PPI.

**Method:**

From 90 PSG recordings, PWA and PPI were derived from finger PPG. We extracted statistical PWA features to capture amplitude drops, and spectral powers to represent pulse amplitude variability. PPI features included statistics and heart rate variability measures. Support vector machine classifiers with different combinations of PWA, PPI, and SpO2 features were trained to detect 60-second segments with apnea or hypopnea. Performance was assessed at both per-segment and per-subject levels for identifying AHI ≥ 15.

**Results:**

Adding PWA to SpO2 improved sensitivity for arousal-related OSA segments from 61.6% to 65.2% and increased per-subject sensitivity from 61.4% to 64.9%. Adding PPI to SpO2 improved per-segment sensitivity for arousal-related OSA segments to 73.3% and increased per-subject sensitivity to 77.2%. Combining PWA with PPI achieved the highest sensitivity for arousal-related segments (77.1%), and when both were added to SpO2, per-subject sensitivity for detecting AHI ≥ 15 reached 80.7%.

**Conclusion:**

Both PWA and PPI improved detection of arousal-related segments and contributed to detecting subjects with AHI ≥ 15. However, PPI consistently outperformed PWA. SpO2 remained particularly important for identifying subjects with AHI < 15.

## Introduction

Obstructive sleep apnea (OSA) is characterized by recurrent upper airway collapse during sleep, leading to intermittent hypoxemia and repeated arousals [1]. The increased inspiratory effort against a closed airway elevates intrathoracic pressure, strains the heart, and triggers sympathetic surges that raise heart rate and blood pressure [2]. Over time, these repetitive events contribute to the development and progression of cardiovascular diseases such as hypertension, stroke, heart failure, and coronary artery disease, as well as metabolic dysfunction and neurocognitive impairment [3, 4]. The consequences of undiagnosed and untreated OSA are medically serious and economically costly.

For decades, polysomnography (PSG) has been the gold standard for OSA diagnosis [5]. Diagnosis is based on the average number of apnea and hypopnea events per hour of sleep, known as the apnea-hypopnea index (AHI). The severity of OSA is classified as mild (AHI ≥ 5–15), moderate (AHI ≥ 15–30), and severe (AHI > 30) [5]. However, because PSG is also used to diagnose other sleep-related disorders and requires manual sleep scoring by well-trained sleep technicians, it often involves long wait times and is inconvenient for routine screening or ruling out OSA.

As a result, alternative approaches such as home sleep apnea testing (HSAT) and wearable devices have gained increasing attention as more accessible and scalable solutions for OSA screening [6–10]. Among the physiological signals available in these devices, oxygen saturation (SpO2) measured by pulse oximeter remains the most promising channel, especially in pediatric patients [8–10]. SpO2 reflects the arterial oxygen content and allows detection of intermittent hypoxemia, which is a hallmark of obstructive sleep apnea. Apnea events typically cause larger and more prolonged desaturations than hypopneas, and longer respiratory events correlate closely with both the depth and duration of desaturation [11]. However, not all hypopnea events are accompanied by desaturations. In accordance with the AASM standards, hypopnea events may also be defined by a reduction in airflow ≥ 30% accompanied by a cortical arousal, even in the absence of significant oxygen desaturation. Relying solely on desaturation-based features may therefore miss moderate or severe OSA with frequent non-desaturating hypopnea events.

To address this limitation, previous research has utilized additional signals derived from the raw photoplethysmography (PPG) waveform, which is recorded by the same pulse oximeter sensor that provides SpO2 measurements. One widely used signal is the pulse-to-pulse interval (PPI) of PPG. PPI reflects the timing between successive pulses and is determined by the cardiac cycle. During cortical arousals, PPI shortens and shows characteristic changes similar to those observed in heart rate [12]. Moreover, pulse-to-pulse variability, or pulse rate variability (PRV) has also been used as a surrogate for heart rate variability in many OSA detection tasks [13–15].

While PPI and PRV have been utilized in many studies, far less attention has been given to pulse wave amplitude (PWA), another PPG-derived signal that reflects changes in vascular tone. PWA drops indicate transient peripheral vasoconstriction triggered by sympathetic activation during cortical arousals [10, 16, 17]. Also, a recent study demonstrated that the magnitude of PWA drops can distinguish between hypopnea with and without desaturations [17]. Beyond PWA drops, pulse amplitude variability (PWAV) provides a measure of peripheral sympathetic modulation [18, 19]. Lower PWAV has been linked to severe OSA with elevated cardiovascular risk [20].

PWA drops and PWAV may capture arousal-related changes that are not fully expressed in heart rate responses. To date, most studies have relied solely on SpO2 or PPI signals for OSA detection, and evidence on the utility of PWA is limited. In this study, we evaluated PWA, PPI, and SpO2 signals from a pulse oximeter to determine whether incorporating PWA features could improve pulse oximetry-based screening performance, and to compare its performance with that of PPI.

## Methods

### Hospital dataset

This was a retrospective study where 316 PPG and SpO2 recordings from full-night PSG studies performed between October 2022 and March 2023 at the Sleep Laboratory Center of Songklanagarind Hospital were initially screened. The PSG studies were conducted as part of routine clinical evaluation for suspected OSA in adult patients.

To ensure balanced representation between sexes, eligible recordings were ordered by date and alternately selected between male and female subjects, without stratification by OSA severity. The inclusion criteria were: patients undergoing first-time diagnostic PSG, aged 18–65 years, and without known cardiac arrhythmias. PSG studies were excluded if the total sleep time was less than 4 h or if the PPG signal quality was insufficient for reliable pulse detection, such that individual pulses could not be consistently identified for analysis. This resulted in the selection of 90 PSG studies for analysis.

In addition, annotations of sleep stages and respiratory events were collected as references for re-labeling data segments for classification purposes and for the final evaluation of the models. Sleep stages and respiratory events were scored by certified sleep technicians and board-certified sleep specialists according to the 2020 American Academy of Sleep Medicine (AASM) manual. The severity of OSA was determined according to standard AHI thresholds: no OSA for AHI < 5, mild OSA for 5 ≤ AHI < 15, moderate OSA for 15 ≤ AHI ≤ 30, and severe OSA for AHI > 30 [21].

This study protocol was approved by the Human Research Ethics Committee, Faculty of Medicine, Prince of Songkla University (REC 66-019-38-2).

### Data processing

PPG and SpO2 signals were acquired from the Compumedics Profusion PSG system using a Nonin 8000JFW pulse oximeter sensor. PPG signals were recorded via the plethysmographic channel at an original sampling rate of 1024 Hz, capturing only the AC component of the waveform. PPG signals were band-passed (0.7–20 Hz Butterworth) and resampled to 128 Hz for efficient analysis. Artifact rejection was performed on 60-second windows to remove unusually large PPG values, identified as exceeding 1.5 times the window standard deviation. The SpO2 signal, derived from the same sensor, was recorded at a sampling rate of 16 Hz and used at its native resolution for further analysis.

**Fig. 1:**
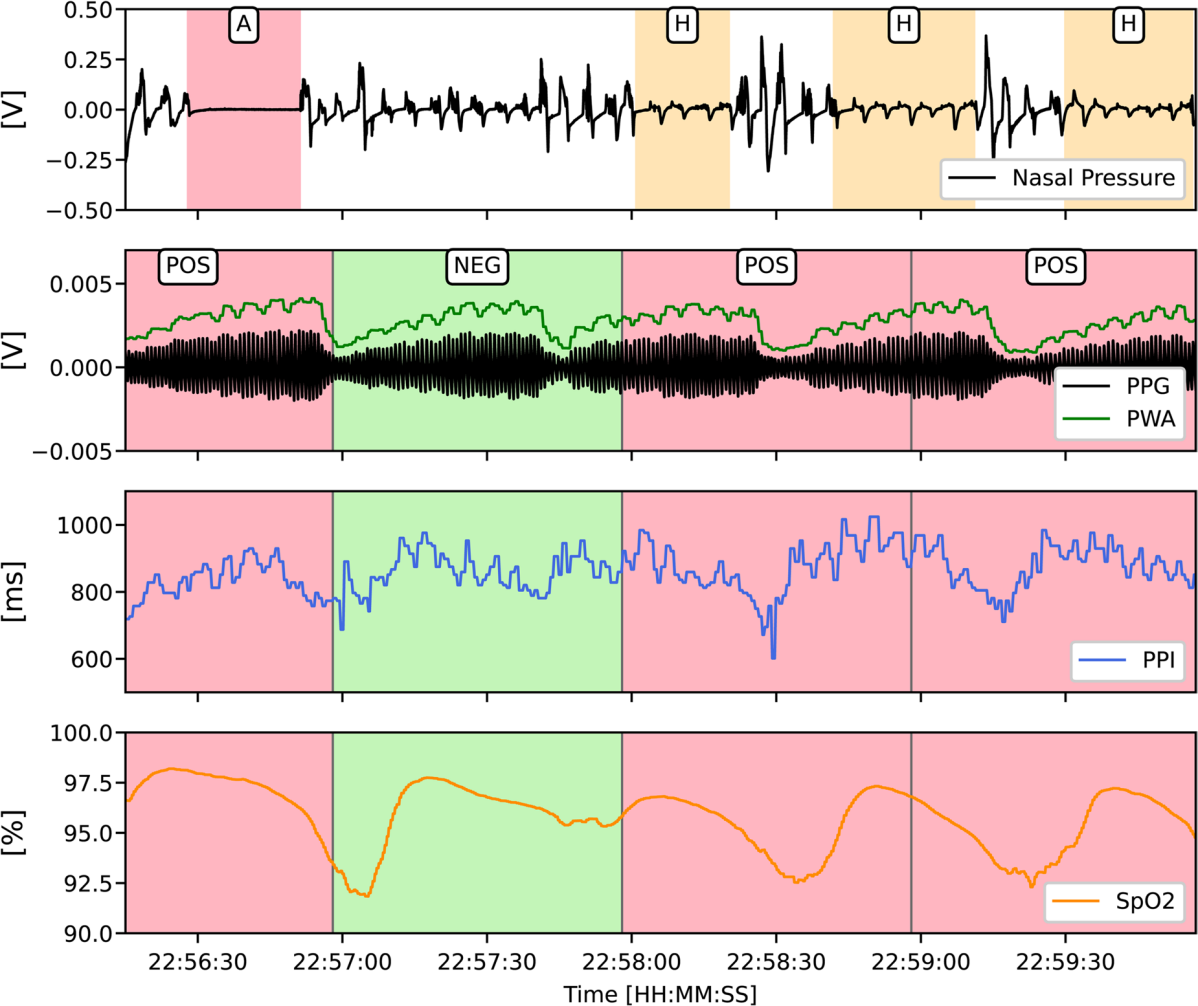
Example of PPG-derived signals during respiratory events and 60-second segment labeling. Top panel: Nasal pressure signal with annotated apnea (**A**) and hypopnea (**H**) events. Panels 2–4: PPG with pulse wave amplitude (PWA), pulse-to-pulse interval (PPI), and oxygen saturation (SpO2). Segment labels: red = positive, green = negative

PPG and SpO2 signals were divided into non-overlapping 60-second segments. This window length was chosen to ensure sufficient time to capture delayed cardiovascular responses, such as pulse rate and vasoconstriction changes, that may persist after the termination of a respiratory event. Each segment was labeled positive if an apnea or hypopnea event occupied at least 10 s of the window; otherwise, it was labeled negative (Fig. 1). From PPG, we extracted features from PPI and PWA. PPI was defined as the time interval between successive systolic peaks, while PWA was computed as the amplitude difference between each peak and its associated trough. To enable frequency-domain analysis, both PPI and PWA sequences were resampled to a uniformly sampled time series at 4 Hz.

### Feature extraction

From predefined segments, features were subsequently extracted from PPI, PWA, and SpO2 signals. Each segment was thus associated with a corresponding feature set and a binary label (positive or negative). To characterize PRV, we extracted time- and frequency-domain parameters typically derived from R-to-R intervals, but in our case computed from the PPI signal. Time-domain parameters included metrics such as the root mean square of successive differences (RMSSD), standard deviation of normal-to-normal intervals (SDNN), and coefficient of variation of successive differences (CVSD) [22]. Frequency-domain features included band-power in the very-low-frequency (VLF: 0.003–0.04 Hz), low-frequency (LF: 0.04–0.15 Hz), and high-frequency (HF: 0.15–0.4 Hz) ranges, computed using the Welch method [23]. In addition, we calculated entropy-based measures, which assess the irregularity and complexity of the signal by quantifying how often short patterns in the data are repeated. Low entropy indicates more regular waveforms, while high entropy indicates higher irregularity. We calculated three entropy measures—sample entropy, dispersion entropy, and fuzzy entropy—each defining pattern similarity differently. They were selected based on their contribution to ECG-based OSA detection in previous work [24].

To characterize changes in PWA, we extracted time-domain features such as minimum, maximum, standard deviation, and coefficient of variation. For PWAV, we computed spectral powers in the frequency ranges associated with autonomic modulation as in PRV analysis, but applied to the PWA signal [18, 19]. All PWA-related features were normalized per subject using min-max scaling, allowing changes in PWA features to be compared across subjects without imposing predefined thresholds. 

For SpO2, only entropy-based features and statistical features were extracted, mainly to capture variability in the SpO2 signal during desaturation events. A full list of extracted features is provided in Table 1.

**Table 1:** Summary of 37 features extracted from PWA, PPI and SpO2 signals

Signal	Domain	Feature	Description
PPI	Time-domain	Mean_NN_	Mean of NN intervals (artifact-free pulse-to pulse-intervals)
		Max_PR_, Min_PR_	Maximum and minimum values of pulse rate
		SDNN, CVNNI	Standard deviation and coefficient of variation of NN of successive NN differences
		SDSD, CVSD	Standard deviation and coefficient of variation of successive NN differences
		NN50, pNN50	Count and percentage of successive NN longer than 50 ms
		NN20, pNN20	Count and percentage of successive NN longer than 20 ms
		RMSSD	Root mean square of successive differences between NN
		Range_NN_	Range of NN
	Nonlinear	Samp_NN_, Fuzz_NN_, Disp_NN_	Sample entropy, fuzzy entropy, and dispersion entropy of pulse intervals
	Frequency-domain	VLF, LF, HF	Spectral powers of pulse intervals in very low, low and high frequency bands
		LFnu, HFnu, LF/HF	Normalized LF and HF power, and their ratio
		SpecEnt	Spectral entropy of PPI signal
PWA	Time-domain	Max_PWA_, Min_PWA_	Maximum and Minimum of pulse wave amplitude
		Std_PWA_, CV_PWA_	Standard deviation and coefficient of variation of pulse wave amplitude
	Frequency-domain	VLF_PWA_, LF_PWA_, HF_PWA_	Spectral powers of pulse wave amplitude in very low, low and high frequency bands
SpO2	Time-domain	Min_Sp_, Mean_Sp_	Minimum and Mean values of oxygen saturation
		Std_Sp_, Var_Sp_	Standard deviation and variance of oxygen saturation
	Nonlinear	Samp_Sp_, Fuzz_Sp_, Disp_Sp_	Sample entropy, fuzzy entropy, and dispersion entropy of oxygen saturation

### Model development and performance evaluation

A support vector machine (SVM) classifier was implemented to classify between positive and negative segments. SVM is a kernel-based method that projects features into higher-dimensional spaces to identify a decision boundary that best separates between classes [25]. We trained five SVM classifiers using five feature combinations: (1) SpO2 only, (2) PWA + SpO2, (3) PPI + SpO2, (4) PWA + PPI, and (5) PWA + PPI + SpO2. 

Model selection and feature selection were performed using nested 5-fold cross-validation. In each fold, 72 recordings (80%) were used for training, while the remaining 18 recordings (20%) were held out for testing. Within each training fold, recursive feature elimination with 5-fold cross-validation (RFECV) was applied using a random forest model to identify the optimal feature subset that maximized performance [26]. Hyperparameter tuning for the SVM was then performed via randomized search with another 5-fold cross-validation to select optimal regularization parameters from {0.1, 1}, and kernel types from {RBF, polynomial}.

To address the class imbalance between positive and negative segments, random undersampling was applied within each training fold prior to model fitting. Classification performance was evaluated at the segment level using accuracy, sensitivity, specificity, precision, and the area under the receiver operating characteristic curve (AUC) [27]. For per-subject screening, the predicted AHI was computed by counting the number of segments classified as positive and dividing that number by the total recording time (hours). Subjects were then classified as positive for moderate-to-severe OSA if their estimated AHI ≥ 15 and negative otherwise (AHI < 15). Per-subject sensitivity was computed as the proportion of the true positive subjects correctly identified by the model. Per-subject specificity was computed as the proportion of true negative subjects correctly identified by the model.

## Results

### Subject characteristics

A total of 90 Type I PSG recordings were analyzed, comprising an even distribution of male (48.9%) and female (51.1%) subjects (Table 2). The average age was 42.5 ± 12.9 years, and the mean BMI was 30.8 ± 7.8 kg/m², indicating a predominantly overweight population. On average, participants reported mild excessive daytime sleepiness based on Epworth sleepiness score (ESS).

**Table 2:** Baseline characteristics

Category	*N* = 90
Male	44 (48.9%)
Age (years)	42.5 ± 12.9
BMI (kg/m^2^)	30.8 ± 7.8
ESS	9.45 ± 4.7
Recording time (h)	7.4 ± 1.3
Total sleep time (h)	5.9 ± 1.6
AHI < 5	11 (12.2%)
5 ≤ AHI < 15	22 (24.4%)
15 ≤ AHI ≤ 30	32 (35.6%)
AHI > 30	25 (27.8%)
*Event composition*	
Obstructive apnea (%)	13.6 ± 19.7
Hypopnea (%)	84.5 ± 41.2
Sleep efficiency (%)	79.65 ± 15.5
Stage N1 (%)	21.7 ± 13.5
Stage N2 (%)	47.6 ± 10.7
Stage N3 (%)	14.4 ± 8.4
Stage REM (%)	16.2 ± 8.6
*Comorbidities*	
Dyslipidemia	27 (30.0%)
Hypertension	26 (28.9%)
Diabetes	12 (13.3%)

Values are presented as means ± SD or numbers (%)

If grouped by severity threshold, 33 subjects (36.6%) had AHI < 15 and 57 subjects (63.3%) had AHI ≥ 15. A majority of participants had moderate to severe OSA. Respiratory events were predominantly hypopneas across all subjects, with apneas accounting for only 13.6 ± 19.7% of events on average. Regarding comorbidities, 30.0% of participants had dyslipidemia, 28.9% had hypertension, and 13.3% had diabetes.

### Per-segment classification using PWA, PPI and SPO2-based features

Table 3 displays the distribution of positive and negative segments from the test folds. Segments with arousals are those in which at least one cortical or spontaneous arousal was ongoing. On average, each test fold comprised 6747.4 segments, of which 1550.4 were positive and 5197.0 were negative, corresponding to 22% and 78%, respectively. Among positive segments, 49.8% were associated with arousals, compared to 12.5% of the negative segments.

**Table 3:** Average number of positive and negative segments across 5 test folds (means ± SD)

Segment type	Total segments	With arousals	No arousals	% With arousals
Positive	1550.4 ± 62.7	771.2 ± 48.6	303.8 ± 52.3	49.8 ± 4.5
Negative	5197.0 ± 125.5	652.2 ± 39.5	4544.8 ± 99.8	12.5 ± 0.6
Total	6747.4 ± 1924.4	1423.4 ± 21.3	4848.6 ± 135.6	21.1 ± 0.8

Table 4 displays the classification performance for all 60-second segments on these test folds. Using only SpO2 features achieved an accuracy of 79.6%, sensitivity of 75.8%, and specificity of 82.8%. Incorporating PWA features into the SpO2 model did not alter the classification performance. In contrast, combining PPI features with the SpO2 model increased sensitivity from 75.8% to 78.3% (*p* = 0.028), and AUC from 0.84 to 0.87 (*p* = 0.004). Combining all three feature sets produced nearly identical performance to the PPI+SpO2 model (accuracy 80.7% vs. 80.6%, sensitivity 78.8% vs. 78.3%, AUC 0.87 for both). Across all models, precision was the lowest among the reported metrics, with the PWA + PPI model achieving the lowest score (65.8%), indicating a higher proportion of false positives relative to true positives.

**Table 4:** Average per-segment classification performance across three feature sets (means ± SD)

Feature Set	No. of Features	5-fold Cross Validation				
		Acc (%)	Sen (%)	Spec (%)	Pre (%)	AUC
SpO2	4.6 ± 3.1	79.6 ± 3.1	75.8 ± 2.4	82.8 ± 5.8	72.7 ± 2.5	0.84 ± 0.02
PWA+SpO2	11.2 ± 1.5	79.8 ± 0.8	75.6 ± 1.3	83.1 ± 1.7	72.2 ± 1.0	0.84 ± 0.02
PPI+SpO2	15.0 ± 1.7	80.6 ± 1.7	78.3 ± 1.8*	82.5 ± 1.8	73.6 ± 1.9	0.87 ± 0.02**
PWA + PPI	10.4 ± 1.5	71.5 ± 1.5*	70.8 ± 1.7*	72.2 ± 3.0	65.8 ± 1.1	0.77 ± 0.02***
PWA + PPI+SpO2	24.0 ± 1.3	80.7 ± 1.3	78.8 ± 1.4*	82.4 ± 1.4	73.9 ± 1.4	0.87 ± 0.01*

* *p* < 0.05, ** *p* < 0.01, *** *p* < 0.0001 after Bonferroni-corrected paired t-tests following repeated-measures ANOVA (comparisons against the SpO2 model)

**Fig. 2:**
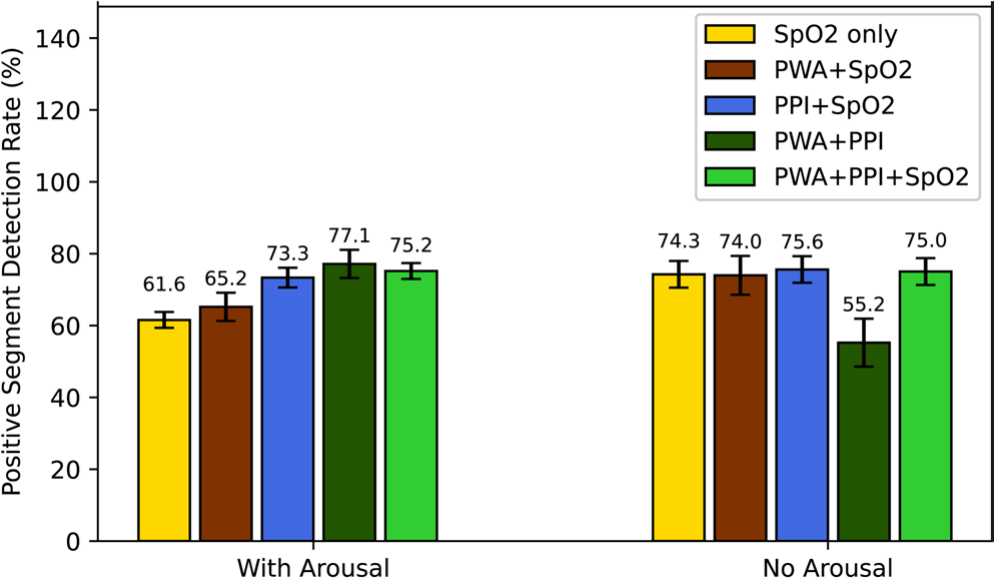
Percentage of positive segments detected by each model

To assess the contribution of PWA and PPI to detection performance in relation to arousals, we compared the proportion of positive segments with arousals correctly detected in two scenarios: with arousals and without arousals (Fig. 2). When arousals were present, the SpO2 model showed the lowest detection rate (61.6%). Adding PWA features improved the detection rate to 65.2%. Incorporating PPI features increased the detection rate to 73.3%. When both PWA and PPI features were combined, the model achieved the highest detection rate of 77.1%, followed by the PWA + PPI+SpO2 feature set (75.2%). In contrast, when no arousals were present, the detection rate dropped to 55.2% in the PWA + PPI model, whereas the models including SpO2 achieved similar detection performance (74.0–75.6%).

**Fig. 3:**
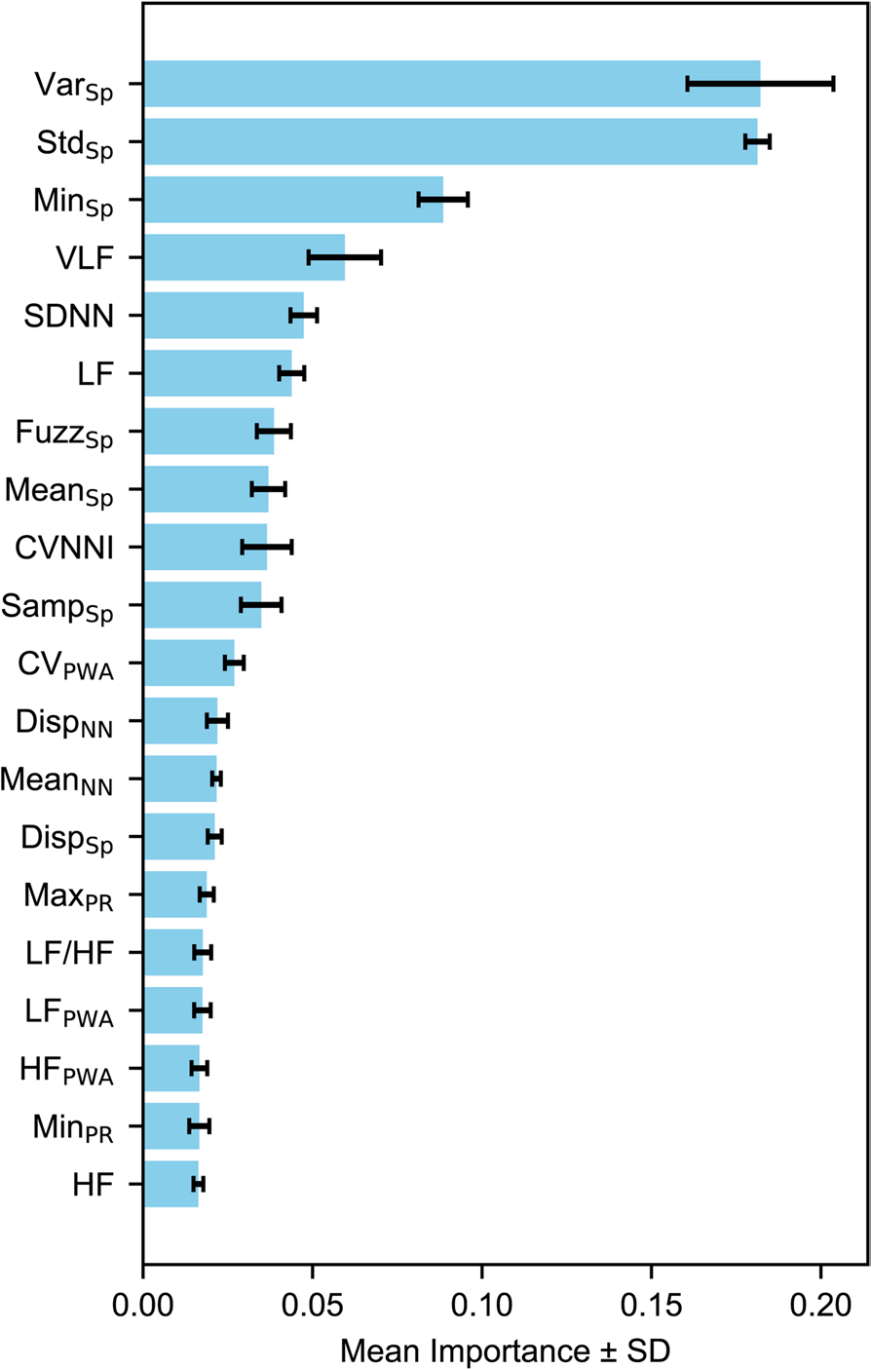
Top 20 features ranked by mean importance from the random forest model

To identify which features contributed most to model performance, we examined average feature importance across five folds (Fig. 3). The top three features were Var_sp_, Std_sp_, and Min_sp_, all derived from SpO2. Pulse rate variability features such as VLF, SDNN, LF, CVNNI, and LF/HF were ranked higher in importance than any PWAV-related features, suggesting that they had a stronger influence than vasoconstriction-related amplitude changes. The only PWA-derived feature with notable influence was the coefficient of variation of PWA (CV_PWA_), whereas amplitude-based features such as Min_PWA,_ and Max_PWA_ did not rank among the top 20 features.

### AHI Estimation and OSA screening

**Fig. 4:**
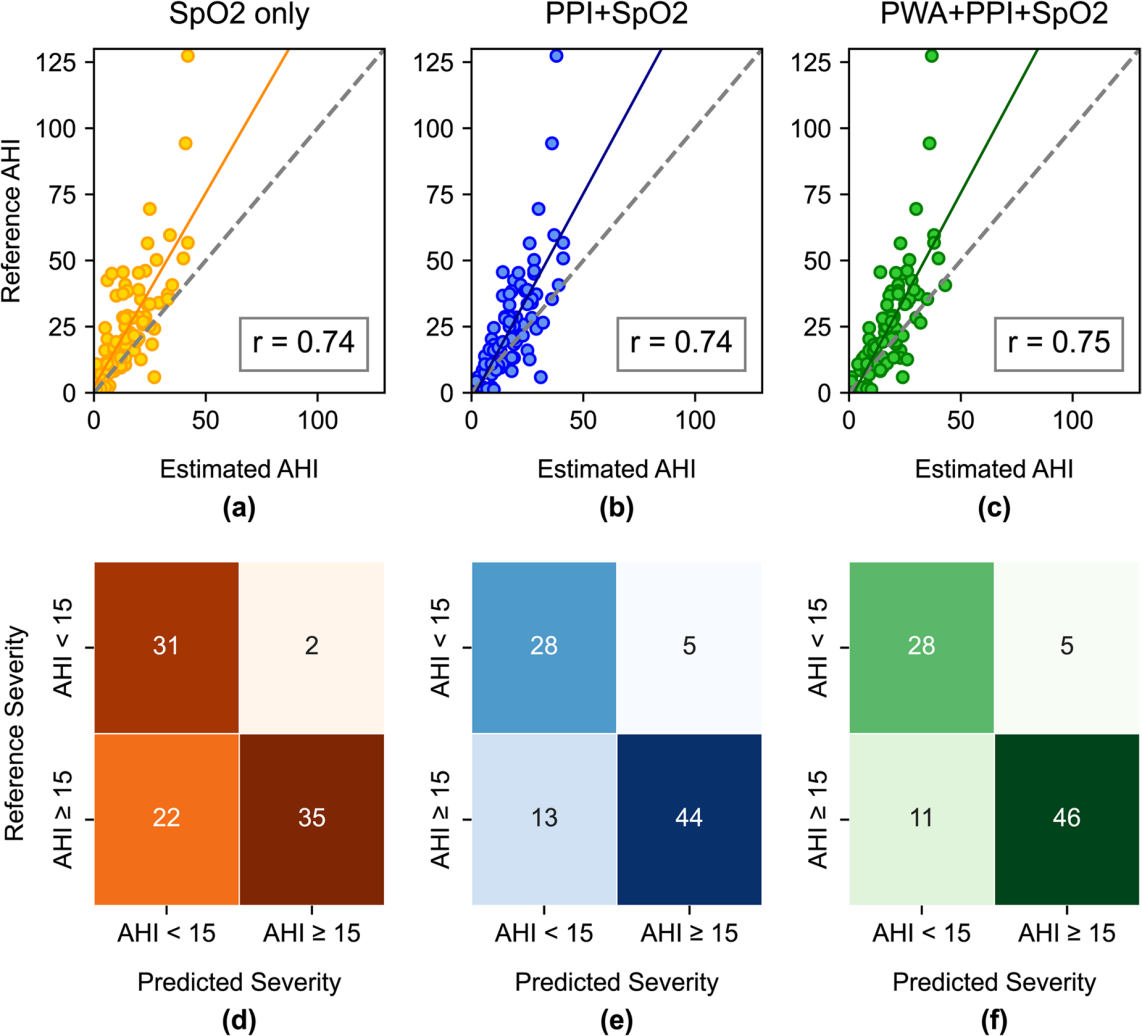
(**a**–**c**) Scatter plots showing the correlation between estimated AHI and reference AHI: r denotes the Pearson correlation coefficient. (**d**-**f**) Per subject confusion matrices for classifying subjects with AHI ≥ 15

Figure 4a-c show AHI estimates from the three best-performing feature sets in per-segment detection – the SpO2 only, PPI+SpO2, and PWA + PPI+SpO2 models. The AHI values were computed based on the average number of positive segments per hour. We found that the estimated AHI from the three models had a strong correlation with the reference AHI (*r* = 0.74–0.75); however, all models tended to underestimate the true AHI values. For per-subject screening, classification was based solely on whether the estimated AHI ≥ 15.

Figure 4d-f represent the confusion matrices, summing the number of subjects correctly and incorrectly classified as having AHI ≥ 15 and AHI < 15. Clearly, incorporating PPI features improved screening for moderate-to-severe OSA (AHI ≥ 15) by detecting 44 out of 57 subjects – 9 more subjects compared to the SpO2 only model (Fig. 4e). Adding PWA features correctly classified two more subjects with AHI ≥ 15 (Fig. 4f), achieving screening sensitivity of 80.7% compared to 77.2% with the PPI+SpO2 model (Table 5).

**Table 5:** Screening performance for subjects with AHI ≥ 15

Subject group	Feature Set	Sen (%) AHI ≥ 15	Spec (%) AHI ≥ 15
All subjects (*n* = 90)	SpO2	61.4	**93.9**
	PWA+SpO2	64.9	81.8
	PPI+SpO2	77.2	84.8
	PWA + PPI	**80.7**	39.4
	PWA + PPI+SpO2	**80.7**	84.8
With Comorbidities (*n* = 45)	SpO2	61.8	**100**
	PWA+SpO2	64.7	81.8
	PPI+SpO2	70.6	90.9
	PWA + PPI	**76.5**	45.4
	PWA + PPI+SpO2	**76.5**	81.8

The ability to correctly identify subjects with AHI < 15 or specificity appeared to be driven primarily by the SpO2 features. The SpO2-only model achieved the highest specificity (93.9%), correctly ruling out 31 of 33 subjects with no or mild OSA (Table 5; Fig. 4d). When combining SpO2 features with PWA or PPI, the specificity decreased to 84.8% in the PPI+SpO2 and PWA + PPI+SpO2 models (Table 5). Moreover, the model incorporating only PWA and PPI features achieved a specificity of 39.4%.

When the screening performance was stratified by the presence of comorbidities (dyslipidemia, hypertension, or diabetes), 33 out of 45 subjects had AHI ≥ 15. The screening performance in this subgroup was lower than in the overall cohort. Nonetheless, similar trends across feature sets were observed. The per-subject sensitivity increased from 61.8% to 70.6% when PPI features were included. Adding PWA identified two additional subjects with AHI ≥ 15 and improved per-subject sensitivity to 76.5%.

## Discussion

This study evaluated whether incorporating PWA features into a pulse oximetry-based machine learning model could improve per-segment detection and enhance OSA screening performance compared to models using only SpO2 or PPI features. In per-segment classification, PWA improved detection of arousal-related OSA segments, but the gain was smaller than with PPI. Previous work has shown that the PWA drops and PWAV are strongest following apnea with desaturation [17]. Combining apneas and hypopneas into a single positive class may have obscured specific PWA patterns, limiting the model’s ability to set an optimal threshold for distinguishing positive from negative segments.

Prior studies have used manually-defined thresholds to define PWA drops (30–50% from baseline) to detect arousals in children and adults [9, 10, 16]. However, such cutoffs are less practical for automatic detection, particularly for entirely new datasets. In our analysis, PWA features were normalized within each subject using its own minimum and maximum values to account for inter-individual variability. The same approach was applied to PPI features. However, most PPI features were still ranked higher than PWA features, and variables such as minimum PWA ranked lower than mean PPI and maximum pulse rate. The difference may be attributed to within-subject variations in PPG signal magnitude, which can arise from changes in sensor contact overnight. Although PWA is expected to provide complementary information and improve prediction performance, its amplitude-based nature makes it more sensitive to such variability than PPI, which reflects beat-to-beat timing. Consequently, PWA changes in response to autonomic drives associated with respiratory events may be less consistent within individuals.

The PWA spectrum likely reflects peripheral sympathetic modulation, potentially mediated in part by baroreflex, as evidenced by a strong coherence between the low-frequency components of PPG and blood pressure [18, 19]. However, that may not be the only mechanism driving PPG oscillations. PPG also captures local vascular influences such as endothelial-mediated tone changes or myogenic activity [28], which we did not adjust for. Thus, these factors may have confounded the PWA spectral features. Future studies could mitigate these effects by scaling PWA spectral powers using the non-pulsatile (baseline) component of the PPG signal.

Incorporating PPI features into the SpO2 model substantially improved detection for subjects with AHI ≥ 15. In our dataset, arousals occurred more frequently in moderate-to-severe OSA, which likely explains the benefit of incorporating arousal-sensitive features, as higher AHI and arousal frequency are both indicative of OSA severity [29]. Although including PWA with PPI resulted in only two additional detections, this may still be clinically meaningful in large-scale or home-based OSA screening, as these additional detections may reflect improved identification of hypopnea-dominant cases that are less well captured by SpO2-only approaches. Notably, this improvement can be achieved without additional sensors, cost, or patient burden, as PWA features are derived from the same pulse oximetry signal. These findings suggest the potential complementary value of PWA that warrants further exploration, particularly in subgroups with pronounced vascular responses during arousals.

Reduced screening sensitivity and specificity in the comorbidity subgroup may reflect altered physiological responses. Hypertension and dyslipidemia can blunt PPG through heightened sympathetic tone and vascular stiffness [30, 31]. These alterations may diminish the magnitude of arousal-related changes, making it harder for the model to recognize those events.

The present study has several limitations. Combining apnea and hypopnea into a single class may have limited the model’s ability to learn subtype-specific physiological patterns. Separating hypopneas with arousals from those without could have allowed PWA features to contribute more to model performance. Clusters of events may also elicit stronger cardiovascular responses, whereas isolated events may produce minimal responses. When using fixed-length segments, this variability can lead to overestimation in some cases and underestimation in others. Lastly, we did not account for sleep stages, which are known to influence arousal occurrences. Although PWA contributed only marginal improvements, our findings underscore the feasibility of leveraging pulse oximetry–derived features beyond SpO2. Such PPG-based approaches can be implemented in the home setting and can help prioritize suspected OSA patients for full polysomnographic evaluation.

## Data Availability

The data that support the findings of this study are available from the corresponding author upon reasonable request.
